# Brucellosis in Takins, China

**DOI:** 10.3201/eid1809.120069

**Published:** 2012-09

**Authors:** Jing Luo, Zhigao Zeng, Yanling Song, Hongxuan He

**Affiliations:** Chinese Academy of Sciences, Beijing, People’s Republic of China

**Keywords:** brucellosis, *Brucella melitensis*, takin, *Budorcas taxicolor*, bacteria, zoonoses, China

**To the Editor:** Brucellosis is a highly contagious bacterial disease and one of the world’s major zoonoses. It is responsible for enormous economic losses in livestock, and it threatens human health and wildlife populations (*1*). In most host species, brucellosis primarily affects the reproductive system, leading to concomitant loss in productivity of affected animals (*1*). Brucellae have been found in wildlife, such as bison, elk, and wild boar, potentially posing a threat for zoonosis (*2*). Currently, the genus *Brucella* comprises 10 species, which are divided according to host specificity and ability to cause chronic infections in human and animals (*3*,*4*). Most *Brucella* species are associated primarily with certain hosts, presumably the result of evolutionary adaptation to a successful host. *Brucella melitensis* is the species most pathogenic in humans and the species most commonly involved in ovine and caprine brucellosis.

In January 2009, in the nature reserve in Qinling Mountains, China, hygromas were found on the knees, stifles, hocks, haunches, and bursae between the nuchal ligament and the primary thoracic spines of 10 free-ranging takins (*Budorcas taxicolor*). The hygroma contents and tissue samples were collected by using aseptic technique, packed separately, cooled immediately, and stored frozen at −20°C until cultured. The samples were streaked onto blood agar and MacConkey agar and incubated aerobically or anaerobically with 5% CO_2_ at 37°C for 4 days.

Tiny gram-negative coccobacilli were isolated. The organism was nonmotile at 20°C and 37°C, and it stained red with the Stamp modification of the Ziehl-Neelsen method. The organism was identified as *B. melitensis* by the Vitek 2 GN identification system (bioMérieux, Marcy l’Étoile, France). The isolate was urease positive, catalase positive, and oxidase positive. It did not require carbon dioxide for growth and did not produce hydrogen sulfide. The isolate could be agglutinated by A-monospecific antiserum but not by M-monospecific antiserum or rough *Brucella-*specific antiserum. It was sensitive to Berkeley and Iz phages at routine test dilution but not sensitive to Tbilisi, Weybridge, Firenze, and R/C phages. According to classical biotyping methods, the isolate was identified as *B. melitensis* biotype 2 (*5*).

Molecular identification by 16S rRNA gene sequencing was used in this study (*6*). According to nucleotide–nucleotide GenBank search by using BLAST (http://blast.ncbi.nlm.nih.gov/), the sequence was 100% identical to the sequences of 16S rDNA of brucellae, especially reference strains including *B. melitensis* 16M (GenBank accession no. NC_003317), *B. abortus* biovar 1 str. 9–941 (NC_006932), *B. suis* 1330 (NC_004310), *B. canis* American Type Culture Collection 23365 (NC_010103), and *B. ovis* American Type Culture Collection 25840 (NC_009505). The isolate was further confirmed as *B. melitensis* according to the 731-bp product by using AMOS-PCR, which discriminates among species by the unique locations of the IS*711* element (*7*,*8*). The restriction pattern of the *omp2b* gene by *Hin*f I was accordant with pattern 3 reported by Cloeckaert et al. (*9*); this finding further indicated that the isolate was *B. melitensi*s (*9*).

The takin (*Budorcas taxicolor*) is a ruminant belonging to the family Bovidae, subfamily Caprinae, genus *Budorcas* (Figure). Takins are found in eastern Asia and Southeast Asia and are listed as “vulnerable A2cd” by the International Union for Conservation of Nature (*10*). Brucellosis might pose a major direct or indirect threat to the conservation of endangered species, such as takins, and can be a source of conflicts among stakeholders in conservation efforts.

**Figure:**
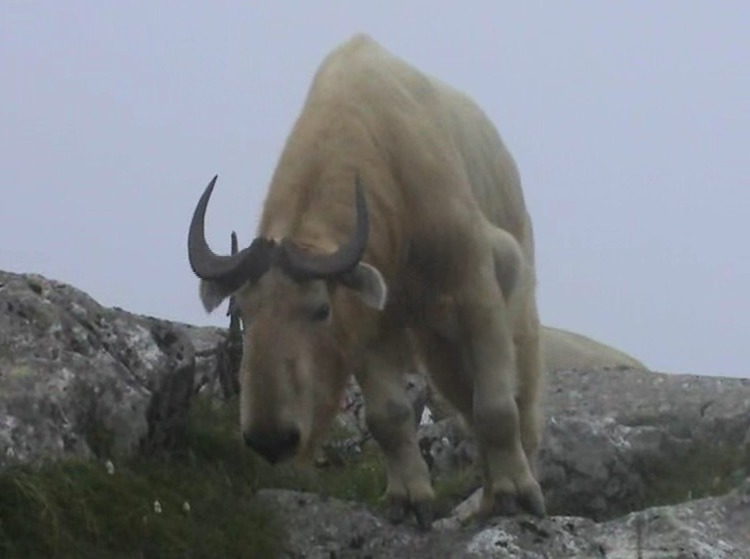
Takin (*Budorcas taxicolor*).

Several antelopes, such as takins, serows (*Capricornis sumatraensis*), and gorals (*Naemorhedus goral*), occur sympatrically in the Qinling Mountains of China. Because brucellae are often transmitted by direct contact or exposure to a contaminated environment, it is possible that rather than being a natural reservoir for the bacteria, takins are infected horizontally by contact with birth exudates from other infected animals (*2*). However, information on brucellosis prevalence in those sympatric ruminants in China is insufficient. Therefore, further investigation and research are needed to test this hypothesis. Also, brucellosis is endemic among livestock and human populations in western China. Because domestic sheep and goats are grazed in the mountains, infections in livestock can spill over into wildlife, such as takins. Brucellosis in humans might also be caused by exposure to infected animals during activities like the handling, skinning, and eviscerating of the carcasses of infected animals.

Whether takins are the reservoir host or an accidental host for *B. melitensis* is still unclear. To further understand the interaction of brucellae among wildlife, domestic animals, and humans, and for purposes of brucellosis management and control, systematic investigations of brucellosis prevalence among wildlife should be conducted.
